# The British Journals, &c.: Medical Jurisprudence and Toxicology

**Published:** 1842-10

**Authors:** 


### MEDICAL JURISPRUDENCE AND TOXICOLOGY.

Case of Suicide. A woman twenty-seven years of age, was committed for theft to the jail of Edinburgh. She was much addicted to drinking, and was apparently ill of delirium tremens "after" admission. That night she complained that somebody was chasing her. She was calm in the morning, and she got a jug, composed of glazed earthenware, containing milk, along with a spoon of iron, for breakfast. The matron saw her at eleven, before going to church, gave her a bible, and desired her to learn the first psalm. She was found, at one o'clock, by the matron, on her return from church, lying dead on the floor, her throat horribly cut, and the fragments of the broken jug lying beside her, and covered with blood. The jug was broken into pieces, some of which were of considerable size, and had very sharp edges and angles.

This case forms one of a class not unfrequent in prisons in which suicide is attempted very shortly after confinement, especially by persons convicted for the first time, accustomed to indulge freely in alcoholic liquors, and strongly predisposed to, if not actually under delirium tremens. It is probable that this woman was really under the influence of the disease now named on the very day of her admission. It is remarkable that she had selected, as the instrument of suicide, the broken fragments of her milk jug, in preference either to the glass of the window, or the iron spoon, which if sharpened on the stone floor of the cell, might have been easily made available to the purpose of self-destruction. Indeed, the latter instrument had been partially used for that end; since the wound after having been originally made with the fragments of the jug, seemed to have been deepened and enlarged by the handle of the spoon having been employed in "boring and pressing aside the parts."—Dr. Spittal, Physician to the Royal Infirmary of Edinb.; *London and Edinb. Month. Journ. of Med. Science,* No. vi. *June,* 1842.

---

Poisoning by Sulphuric Acid. The author was called to a young woman supposed to have swallowed sulphuric acid. He found her lying on the floor, with a black froth issuing from her mouth, her extremities cold, pulse almost imperceptible, breathing labouring and irregular. A quantity of whitening mixed with milk, was copiously administered, to the relief of the patient, who now vomited a quantity of streaky mucus. This was on a Friday.

On the following day, enemata, bloodletting, anodyne and mucilaginous drinks were administered, and great pain occurring over the epigastrium, with symptoms of severe gastritis, eighteen leeches were applied to the abdomen, followed by epithems of hot turpenture. She died easily on the afternoon of this day.

The lips were found excoriated and much blackened; the œsophagus congested and also blackened; the cardiac and pyloric ends of the stomach intensely inflamed, blackened and excoriated; the duodenum slightly affected; the other portions of the intestinal tube distended with flatus; the kidneys much inflamed and *exerting an acid reaction on litmus paper;* the right ovary contained a clot of blood; the uterus contained some purulent or mucous (?) fluid adhering to its mucous membrane.

The author obtained distinct traces of the presence of sulphuric acid, both in the dress and in the kidneys of the patient, by the application of Devergie's test.—Dr. Scoffern; *Lancet,* No. 11. *June* 11, 1842.

---

Copora Lutea. To ascertain the precise structure of one of these bodies, is of importance in a medico-legal point of view. The ovary, like other parts, being liable to disease, its vessels may become varicose, and apoplexy, as it is termed, forms no uncommon appearance in it, consisting of an irregular effusion of blood into its tissues, "varying indefinitely as regards size." Tubercles may also occur in it. Both these morbid conditions may be mistaken for corpora lutea. The following appearances ought always to be present in true corpora lutea; 1. A distinct external envelope, in contact and in union with the stroma of the ovary, but capable of being dissected away from it entire. 2. A solid substance, fleshy-looking, red, pinkish or yellow, divided into a greater or less number of lobuli. 3. An inner membrane or ovisac thickened. 4. A central deposit of granular or other matter, or the remains of it. 5. The general microscopic appearance presented will form a good auxiliary means of diagnosis. The fact of one of the radii reaching as far as the surface of the ovary, is useful as a diagnostic indication, but is not decisive, being occasionally present in the false as well as in the true bodies.—Frank Renaud, Esq., Edinburgh; *London and Edinb. Month. Jour. of Med. Science.* No. vi. *June,* 1842,

